# Risk of adverse treatment outcomes among new pulmonary TB patients co-infected with diabetes in Pakistan: A prospective cohort study

**DOI:** 10.1371/journal.pone.0207148

**Published:** 2018-11-08

**Authors:** Fatima Mukhtar, Zahid A. Butt

**Affiliations:** 1 Department of Community Medicine, Lahore Medical & Dental College, Lahore, Pakistan; 2 Department of Epidemiology & Biostatistics, Health Services Academy, Islamabad, Pakistan; 3 School of Population and Public Health, University of British Columbia, Vancouver, Canada; 4 Department of Epidemiology & Biostatistics, Health Services Academy, Islamabad, Pakistan; Indian Institute of Technology Delhi, INDIA

## Abstract

**Purpose:**

The escalating burden of diabetes in countries tackling high burden of tuberculosis (TB) has adverse implications for co-infected individuals and National TB control efforts. We aimed to study whether there was a difference in treatment outcome among diabetic and non-diabetic pulmonary TB patients and identify the determinants of treatment outcome among the two groups.

**Materials and methods:**

This prospective cohort study recruited new patients of pulmonary tuberculosis (PTB) aged 15 years and above who were diagnosed at and registered with Gulab Devi Chest Hospital, Lahore, Pakistan for anti-tuberculosis treatment (ATT). PTB patients were screened for diabetes using random and fasting blood glucose tests. Diabetic and non-diabetic PTB patients were followed up at second, fifth and sixth month of ATT and 6 months after ATT completion to determine treatment outcome. Multivariate logistic regression analysis was conducted to assess association between various factors and treatment outcome.

**Results:**

Of 614 PTB patients, (*n* = 113 [18%]) were diabetic and (*n* = 501 [82%]) non-diabetic. Final model showed that diabetics were more likely to experience an unfavorable outcome as compared to non-diabetics (adjusted odds ratio [aOR] = 2.70, 95% Confidence Interval [CI] = 1.30 to 5.59). Other predictors of unfavorable outcome included rural residence (aOR = 1.98, 95% CI = 1.14 to 3.47), body mass index less than 18.50 (aOR = 1.89, 95% CI = 1.03 to 3.47) and being a smoker (aOR = 2.03, 95%CI = 1.04 to 3.94).

**Conclusion:**

Our study shows unfavorable treatment outcome among diabetic PTB patients. Integrated models of care with screening/testing and management for diabetes and TB could improve TB treatment outcomes.

## Introduction

The burgeoning epidemic of chronic diseases without the subsequent decrease of infectious diseases provides opportunities for interaction between diseases not seen previously and leads to significant public health consequences [1]. There has been a global increase in the adult diabetic population from 108 million in 1980 to 422 million in 2014 due to ageing population, urbanization and change in lifestyles [2]. This has led to a renewed interest in the co-epidemic of diabetes and tuberculosis, which poses a challenge for both the developed and developing world [3]. It has been documented by studies worldwide that 10–30% of the tuberculosis patients may have diabetes [4]. The frequency of TB in diabetic patients was reported as 7.3% and 14.8% by studies conducted in Indonesia and Turkey, respectively [4,5]. According to a study conducted in Pakistan, the prevalence of tuberculosis in hospitalized diabetic patients was found to be 10-times higher than in non-diabetic patients [6].

Diabetes mellitus increases the risk of tuberculosis by three folds [7]. It alters TB disease presentation and has detrimental effect on treatment outcome. These adverse treatment outcomes include: increased chances of relapse, failure, default and death among co-infected patients [8–10]. Diabetes also slows the clearance of tuberculosis bacteria from the sputum by 5 days [11]. According to an Indonesian study, 22.2% of patients with diabetes and 6.9% without diabetes had positive sputum cultures at the completion of a six month anti-tuberculosis treatment [4]. The relative risk of death was found to be 1.89 among TB patients suffering from diabetes compared to TB patients without diabetes [9].

Pakistan is one of the six countries, which contributes 60% of new TB cases globally. In order to end the global TB epidemic as is laid down in the sustainable development goals to be achieved by 2030, much progress in TB prevention has to be made [12]. Researchers are fearful of diabetes mellitus co-morbidity adversely affecting tuberculosis control in a manner similar to HIV. They also dread its negative impact in achieving a 90% reduction in TB deaths and 80% reduction in incidence of TB by 2030 compared with 2015 rates [13–16]. An expert meeting held in November 2009 at the International Union Against TB and Lung Disease identified research agenda for diabetes and tuberculosis, in which the research question addressing TB treatment outcomes in diabetic and non-diabetic patients was placed second on the list of high priority questions [17].

Pakistan has a high burden of TB in addition to its expanding population of diabetics. According to International Diabetes Federation there are over 7 million diabetics in Pakistan with a national prevalence of 6.9% [18]. Data are lacking regarding the impact of diabetes on tuberculosis treatment outcome in Pakistan. There is a potential for distinct social, cultural, economic and ethnic differences in the effect of DM on TB treatment outcome among co-infected individuals, which have not been previously investigated. It is imperative to generate local evidence in order to take corrective action and inform policy and practice. Hence the diabetes-tuberculosis treatment outcome (DITTO) study; a prospective cohort study was undertaken in Pakistan to estimate the risk of adverse outcomes in diabetic patients who were being treated for tuberculosis. To our knowledge this is the first prospective cohort study undertaken in Pakistan to see the tuberculosis treatment outcome among diabetic patients. This study can be a model for other studies on TB and DM in developing countries due to the unique diabetes screening protocol used to identify PTB diabetic patients.

## Materials and methods

### Study design and participants

The details of study methods have been published previously [19]. Briefly, the DITTO study was a prospective cohort study undertaken in October 2013 at Gulab Devi Chest Hospital (GDH), a tertiary care hospital in Lahore, Pakistan. GDH is one of the oldest and biggest cardiothoracic hospitals in South-Asia, which provides free anti-tuberculosis treatment to patients from all over the country, both from the rural and urban areas and all socio economic strata [20]. The DITTO cohort comprised of new adult (15 years and above) cases of pulmonary tuberculosis, both sputum smear positive and sputum smear negative that were registered with GDH for ATT. New case was a PTB patient who had never taken TB drugs in the past, or had taken TB drugs for less than 4 weeks in the past but was not registered with National Tuberculosis Control Program, Pakistan (NTP) [21]. The diagnosis of PTB was made in line with definitions provided by NTP and World Health Organization (WHO) [21,22]. The treatment regimens adhered to in this study were in accordance with those recommended by WHO and NTP. The recruitment of 614 cases was completed in March 2014. Ethical approval was obtained from the Institutional Ethical Review Committee of Health Services Academy, Islamabad (F. No. 107/2013-IERC/HSA). Permission was also taken from the administration of the Gulab Devi Chest Hospital, Lahore, where data collection was undertaken. All patients gave written informed consent before recruitment in the study.

### Data sources

At baseline cohort members’ detailed contact information was obtained, which was refreshed at every follow up visit. Anthropometric data were collected. Respondents completed an interviewer-administered questionnaire, which collated data on socio-demographics, co-morbidities, lifestyle and behavioural characteristics, clinical presentation of TB, family history of diabetes, adherence to DOTS and glyceamic control among diabetics. At recruitment, PTB patients’ diabetic status was ascertained. Patients who gave a self-report of diabetes were labeled as diabetic and all others were screened with a random blood glucose (RBG) test. Among the known diabetic patients, those below 30 years of age who were on insulin monotherapy and had never used any other anti-diabetic medication were labeled as Type 1 diabetic and all others as Type 2 diabetic [23]. PTB patients having a RBG <110mg/dl (<6.1mM) were labeled as non-diabetic [24]. Patients with RBG ≥110mg/dl (≥ 6.1mM), were made to undergo a fasting blood glucose test (FBG) on their next visit which was scheduled at 2 months of follow up to confirm their diabetic status. Fasting was defined as no caloric intake for at least 8 hours. A fasting plasma glucose value ≥ 126mg/dl (7.0mmol/l) was considered diagnostic of diabetes. The cut-off thresholds used were those laid down by WHO [25,26]. At this first follow up visit, contact details were also reviewed and sputum smear examination was performed. At the second follow up visit scheduled at fifth month, while on ATT in addition to the above, blood sample was drawn to determine glycosylated haemoglobin of diabetic patients. PTB cohort was followed up prospectively at second, fifth and sixth month while on ATT and also at six months after ATT completion to determine treatment outcomes. Standardized treatment outcome definitions of NTP and WHO were adhered to in the study (before the 2013 revision) [21,22].

### Outcomes

We assessed diabetic status of PTB patients with treatment outcomes, which was coded as a dichotomous variable into favourable treatment outcome (patients who were cured and who completed treatment) and unfavourable treatment outcome (patients who defaulted or died, were transferred out, who had treatment failure and who relapsed). In addition to our main exposure variable i.e. diabetic status, the co-variates that were studied included socio-demographic characteristics such as age, gender, education, occupation and income. Lifestyle and behavioural characteristics included smoking status, alcohol consumption status and drug abuse. BMI and history of co-morbidities were also included.

### Statistical analysis

Comparisons were made between socio-demographics, lifestyle and behavioural patterns, clinical presentation and co-morbidities in patients with and without diabetes using the Chi-square or Fisher’s exact tests. We performed logistic regression analysis to determine association between diabetic status, other independent variables and treatment outcome. Odds Ratios (OR) and 95% Confidence Intervals (CI) were calculated. Variables with p≤0.20 in the univariate analysis and biological plausibility were included in multivariate model. Biologically meaningful interactions were assessed. Goodness of fit for the final model was evaluated by using Hosmer and Lemeshow goodness of fit test, with a p-value of > 0.5 considered to be a good fit. Statistical package for Social Sciences version 16 was used for data analysis.

## Results

The assessment of respondents’ diabetic status revealed that 18% (*n* = 113) were diabetic and 82% (*n* = 501) were non-diabetic. Of the diabetics in the PTB cohort, 75% (*n* = 85) were known diabetics and 25% (*n* = 28) were newly diagnosed diabetics. (Table 1) Only one patient was diagnosed as Type 1 diabetic among the known diabetics.

**Table 1:** Socio-demographic, lifestyle and co-morbidity characteristics of 614 new pulmonary tuberculosis patients with (n = 113) or without diabetes mellitus (n = 501) presenting at Gulab Devi Chest Hospital, Lahore.

Variable		All cohort n = 614(%)	PTB with DM n = 113(%)	PTB without DM n = 501(%)
Gender	Male	312(51)	53(47)	259(52)
	Female	302(49)	60(53)	242(48)
Educational status	Illiterate	323(52)	74(65.5)	249(50)
	Primary	84(14)	13(11.5)	71(14)
	Matriculation	146(24)	20(18)	126(25)
	Intermediate	30(5)	5(4)	25(5)
	Bachelors	18(3)	1(1)	17(3)
	Masters and above	13(2)	0(0)	13(3)
Area of residence	Urban	424(69)	84(74)	340(68)
	Rural	190(31)	29(26)	161(32)
Age group	15–19 years	135(22)	1(1)	134(27)
	20–24 years	142(23)	4(3.5)	138(27)
	25–29 years	67(11)	4(3.5)	63(13)
	30–39 years	90(15)	16(14)	74 (15)
	40–49 years	67(11)	30(27)	37(7)
	>50 years	113(18)	58(51)	55(11)
Income Category (Rupees)	Nil*	384(63)	73(65)	311(62)
	<5000	43(7)	5(5)	38(8)
	5100–8000	67(11)	6(5)	61(12)
	8100–11000	54(9)	10(9)	44(9)
	11100–14000	26(4)	7(6)	19(4)
	14100–17000	21(3)	6(5)	15(3)
	>17100	19(3)	6(5)	13(2)
Heart disease	Yes	16(3)	14(12)	2(0.4)
	No	598(97)	99(88)	499(99.6)
Hypertension	Yes	39(6)	26(23)	13(3)
	No	575(94)	87(77)	488(97)
BMI^‡^	Less than 18.50	307(51)	18(17)	289(58)
	18.50–24.99	257(42)	63(58)	194(39)
	25–29.99	27(4)	18(17)	9(2)
	30 and above	17(3)	9(8)	8(1)
Drug use	Yes	9(1)	2(2)	7(1)
	No	605(99)	111(98)	494(99)
Tobacco consumption	Never	442(72)	78(69)	364(73)
	Habitually	85(14)	13(12)	72(14)
	Currently	20(3)	7(6)	13(3)
	Used to	67(11)	15(13)	52(10)
Marital status	Married	344(56)	101(89)	243(48.4)
	Single	267(43.5)	12(11)	255(51)
	Divorced	1(0.2)	0(0)	1(0.2)
	Widowed	2(0.3)	0(0)	2(0.4)

*Income in the form of loans/ help from relatives/extended family/friends

^‡^ Body mass index of 608 patients

In univariate logistic regression analysis, patients with diabetes were more likely to experience an unfavourable outcome than patients without diabetes (OR = 2.6, 95% CI: 1.48 to 4.56). Unfavourable outcome was more likely in PTB patients aged 35–54 years (OR = 2.60, 95% CI: 1.46 to 4.62) and those aged 55 years and above (OR = 2.44, 95% CI: 1.15 to 5.18) as compared to 15–34 year old PTB patients. Patients residing in rural areas (OR = 1.96, 95% CI: 1.16 to 3.31) as compared to urban dwellers had greater likelihood of an unfavourable outcome. Males were more likely than females to have an unfavourable outcome (OR = 1.56, 95% CI: 0.93 to 2.62, p = 0.090) but the result was not statistically significant. Smokers were more likely to have an unfavourable treatment outcome (OR = 2.42, 95% CI: 1.33 to 4.38) than non smokers. Unfavourable outcome was more likely among respondents with no formal education (OR = 1.93, 95% CI: 1.15 to 3.26, p = 0.013) as opposed to those with some form of formal education (Table 2).

**Table 2:** Univariate analysis of favorable and unfavorable treatment outcome among 503 pulmonary tuberculosis patients and its predictors.

	Variables	OR	95% CI	P
**Diabetic status**	Diabetic	2.60	1.48 to 4.56	0.001*
	Non-diabetic	1		
**Age category**	15–34 years	1		
	35–54 years	2.60	1.46 to 4.62	0.001*
	55 years and above	2.44	1.15 to 5.18	0.020*
**Gender**	Male	1.56	0.93 to 2.62	0.090
	Female	1		
**Residential Area**	Urban	1		
	Rural	1.96	1.16 to 3.31	0.011*
**Income**	Nil	1		
	<5000	1.47	0.57 to 3.77	0.422
	5100–8000	0.88	0.36 to 2.20	0.788
	8100–11000	1.19	0.50 to 2.83	0.697
	11100–14000	1.10	0.31 to 3.91	0.879
	14100–17000	1.89	0.59 to 6.02	0.281
	>17100	0.51	0.07 to 3.99	0.521
**Educational status**	Illiterate	1.94	1.15 to 3.26	0.013*
	Literate^@^	1		
**Marital status**	Unmarried	1		
	Married	1.67	0.98 to 2.82	0.057
**Smoker**	Yes	2.42	1.33 to 4.38	0.004*
	No	1		
**Drug Use**	Yes	3.90	0.91 to 16.70	0.067
	No	1		
**Type of PTB**	Positive	1.15	0.69 to 1.92	0.586
	Negative	1		
**Cough> 3 weeks**	Yes	3.97	0.94 to 16.75	0.060
	No	1		
**Prolonged fever**	Yes	1.37	0.56 to 3.32	0.490
	No	1		
**Difficulty in breathing**	Yes	1.68	0.90 to 3.13	0.102
	No	1		
**Blood in sputum**	Yes	1.55	0.90 to 2.66	0.111
	No	1		
**Night sweats**	Yes	1.09	0.65 to 1.82	0.749
	No	1		
**Weight Loss**	Yes	1.34	0.58 to 3.07	0.490
	No	1		
**Hypertension**	Yes	1.34	0.49 to 3.62	0.571
	No	1		
**Heart disease**	Yes	2.15	0.57 to 8.13	0.261
	No	1		
**Asthma**	Yes	2.13	0.42 to 10.77	0.361
	No	1		
**BMI**	18.50–24.99	1		
	Less than 18.50	1.29	0.75 to 2.21	0.367
	25–25.99	1.30	0.36 to 4.75	0.693
	30 and above	1.13	0.24 to 5.32	0.875

^@^Primary, Matriculation, Intermediate, Bachelor and Masters and above

* Significant at a p-value ≤ 0.05

The final multivariate analysis showed that diabetics were more likely to experience an unfavourable outcome as compared to non-diabetics (aOR = 2.70, 95% CI = 1.30 to 5.59, p = 0.008), after adjusting for age, residential background, smoking status and BMI. Compared to urban dwellers the risk of unfavorable outcome was more likely among individuals residing in rural areas (aOR = 1.98, 95% CI = 1.14 to 3.47). Similarly, compared to individuals with BMI between 18.50 to 24.99, unfavourable outcome was more likely among individuals with a BMI less than 18.50 (aOR = 1.89, 95% CI = 1.03 to 3.47) after adjustment. Smokers were more likely to have an unfavourable outcome as compared to non-smokers (aOR = 2.03, 95%CI = 1.04 to 3.93) (Table 3). No statistically significant interactions were observed.

**Table 3:** Multivariate analysis for predictors of favourable and unfavourable treatment outcome among 493^¶^ pulmonary tuberculosis patients.

Variables	Adjusted OR	95% CI	P value
**Diabetic status**			
Diabetic	2.70	1.30 to 5.59	0.008*
Non-diabetic	1		
**Age category(years)**			
15–34	1		
35–54	1.56	0.74 to 3.29	0.239
55 and above	1.51	0.62 to 3.70	0.368
**Residential Area**			
Urban	1		
Rural	1.98	1.14 to 3.47	0.016*
**BMI**^**#**^			
18.50–24.99	1		
Less than 18.50	1.89	1.03 to 3.47	0.041*
25–25.99	0.95	0.24 to 3.69	0.936
30 and above	0.91	0.18 to 4.48	0.906
**Smoker**			
Yes	2.03	1.04 to 3.93	0.037*
No	1		

^#^10 patients had missing BMI values

* Significant at p ≤ 0.05

## Discussion

In this study, among all PTB patients, 18% had diabetes whereas 14% of PTB patients undergoing treatment experienced an unfavorable treatment outcome. Our study showed that diabetic PTB patients were more likely to experience an unfavourable outcome as compared to non-diabetic PTB patients corroborating the findings of previous studies [8,9,27]. Among the 14% (69/503) PTB patients who had an unfavourable TB treatment outcome, 28(6%) died, 10 (2%) defaulted, 11 (2%) experienced failure, 5(1%) were transferred out during the 6 months of ATT while 15/449 (3%) experienced relapse during the 6 months after ATT completion. Relapse and death were significantly associated with diabetic status of the cohort members; however, no significant association was found between the treatment outcomes of default, failure, transferred out, and diabetic status of respondents. (Data not shown)

A study conducted in Taiwan also demonstrated an increased risk of unfavourable treatment outcome among diabetic PTB patients after adjusting for age, sex, smoking, type of case, drug resistance and sputum smear. Although, on the addition of diabetes associated morbidity in the model, researchers found an attenuation of the effect of diabetes on unfavorable treatment outcome, with diabetes associated morbidity significantly associated with unfavorable treatment outcome in multivariate model [28]. This detrimental effect of diabetes associated morbidity on treatment outcome was most probably mediated through the effect of longstanding hyperglyceamia in these patients. We were unable to appraise the association of diabetes-associated morbidity or the duration of diabetes on unfavourable treatment outcome in our study, although we included both variables in our data collection tool. This may be attributed to respondents’ lack of knowledge regarding their disease (diabetes associated morbidity) or an inability to recall, as majority of our diabetic PTB respondents were illiterate and elderly. Additionally, it is likely that our physicians do not communicate and inform patients of their illness, resulting in their low level of awareness [29].

A prospective study conducted in South Korea categorized the treatment outcomes of death and failure as unfavourable and identified predictors of this unfavourable outcome. They reported an aOR of 2.52 (95% CI = 1.27 to 5.01) for diabetes as a predictor of treatment outcome [30]. A systematic review conducted by Baker et al pooled the combined effect of failure and death, and evaluated the role of diabetes on this pooled TB treatment outcome. The 12 studies included in the systematic review with similar treatment outcome yielded a pooled RR of 1.69 (95% CI, 1.36 to 2.12) [9]. Several mechanisms have been proposed for this undesirable effect of diabetes on treatment outcome of TB patients. These include; a change in the pharmacokinetics and pharmacodynamics of TB drugs in diabetic patients [31], the anti-tuberculosis medications employed to fight TB especially Rifampicin, which increases insulin resistance [32] and an impairment of immunity induced by diabetes. Patients with diabetes may have reduced (IFN)-υ interferon, decrease in the activation of alveolar macrophages and change in type 1 cytokine expression, indirectly influencing immune response among diabetic PTB patients [33].

There are some studies, which unlike our results demonstrate no difference in treatment outcome between diabetic and non-diabetic TB patients. A retrospective study undertaken at Saudi Arabia did not find any association between diabetes and treatment outcome among TB patients [34]. This lack of association may be attributed to the retrospective nature of the study. Similarly, Khan et al looked at treatment outcome among diabetic patients who were put on standardized TB treatment. The treatment outcome variable studied was binary consisting of the two options; successful and unsuccessful. No difference was found among the two groups i.e diabetic PTB and non-diabetic PTB regarding treatment outcome [35]. Satyanaray et al have attributed these results to inability of the study to achieve adequate power [36]. Another cohort study conducted in India showed no association of diabetes with treatment outcome among TB patients [37]. This could be explained by the small sample size i.e 100 patients, which were enrolled, out of which 7 were excluded hence 93 patients were followed up for treatment outcomes in the study.

Our study found rural area of residence, having a BMI less than 18.50 and being a smoker as independent predictors of unfavourable treatment outcome. The association of rurality with unfavourable treatment outcomes in our study could be related to delayed access to health care providers or medicine or low socio-economic status as reported previously [38]. Rurality is a known determinant associated with delay in the treatment and diagnosis of TB [39]. Choi et al reported consistent results. The patient factors highlighted by them were diabetes, BMI and patients’ age whereas disease factors included MDR-TB, prior ATT intake by the patient and significant regimen changes [30]. Having a low BMI or nutritional disequilibrium alters the host immune response leading to severe form of TB disease and subsequent poor treatment outcomes [40]. Other determinants of poor treatment outcome among TB patients include lifestyle factors such as smoking, alcohol consumption and drug abuse [30]. There are various mechanisms through which smoking may adversely impact on TB treatment outcome; by altering host defense mechanism, affecting lung structure and function and modifying mechanisms of pathogen clearance [41]. Additionally, smoking has been identified as a predictor of loss to follow up which may indirectly lead to poor treatment outcomes due to non-compliance [42]. Our study did not demonstrate an association of alcohol consumption and drug abuse with unfavourable treatment outcome. This lack of association may be attributed to small numbers as only 5% and 2% of respondents self–reported alcohol consumption and drug abuse respectively. This could be an underestimate considering the social, cultural and religious norms that exist in our country [43].

The strengths of our study included employing a prospective cohort study design. We consecutively enrolled new PTB patients registered for treatment within a program setting. All patients were treated with the same regimen for 6 months with outcomes monitored according to standardized treatment outcome definitions provided by WHO. The exposure status of patients was determined by data collection team at study initiation in contrast to other studies, which rely on medical records. The diagnosis of DM was not based on blood glucose levels alone, which would have missed subjects who were euglyceamic at the time of screening i.e the known diabetics whose blood sugar levels were under control. The exposure status of PTB patients’ was based upon two tests; one random and the other fasting blood glucose test. The confirmatory FBG test was conducted two months after initiation of ATT to rule out the bias associated with transient stress induced hyperglycemia attributed to tuberculosis disease.

However, there were certain limitations in this study. Drug susceptibility testing was not done among the PTB cohort at the time of enrollment or during the course of ATT, which could have led to bias in the results. However, because of our inclusion criteria of recruiting only the new PTB patients, with no prior history of ATT intake we hope drug resistance was not an issue. Secondly, HIV status, which has been identified as a strong risk factor for adverse treatment outcome among TB patients, was not determined. Lastly, we were unable to study the effect of glucose control on TB treatment outcome as HbA1c values for the entire cohort were not available. Due to resource constraints glycosylated hemoglobin blood analyses was performed of only the diabetics in the study. If treatment outcome among diabetic PTB patients is modified by glucose control, our results could be affected. However, according to Mi F et al, 2 month and 6 month FBG levels among PTB patients did not have statistically significant association with adverse outcomes [44]. The study results may be extrapolated to program setting with caution, as they may be an underestimate of what would be in the program given the exhaustive follow up of patients undertaken by the study team, which could have enabled favourable outcomes due to adherence and compliance to ATT.

In conclusion, our study shows unfavorable treatment outcome among diabetic PTB patients compared to non-diabetic PTB patients. Our findings suggest linking of TB and diabetes diagnostic and treatment services. Integrated models of care with early screening/testing and management for diabetes and TB should be initiated. The detection of diabetes in TB patients and linking these persons to care may improve TB treatment outcomes.
